# Risk Perceptions of Low Nicotine Cigarettes and Alternative Nicotine Products across Priority Smoking Populations

**DOI:** 10.3390/ijerph18105311

**Published:** 2021-05-17

**Authors:** Rachel L. Denlinger-Apte, Lauren R. Pacek, Jennifer Cornacchione Ross, Maansi Bansal-Travers, Eric C. Donny, Dorothy K. Hatsukami, Dana Mowls Carroll

**Affiliations:** 1 Department of Social Sciences and Health Policy, Division of Public Health Sciences, Wake Forest School of Medicine, Winston-Salem, NC 27157, USA; rdenling@wakehealth.edu (R.L.D.-A.); jcornacc@wakehealth.edu (J.C.R.); 2 Department of Psychiatry and Behavioral Sciences, Duke University School of Medicine, Durham, NC 27705, USA; lauren.pacek@duke.edu; 3 Roswell Park Comprehensive Cancer Center, Department of Health Behavior, Buffalo, NY 14263, USA; maansi.travers@roswellpark.org; 4 Department of Physiology and Pharmacology, Wake Forest School of Medicine, Winston-Salem, NC 27157, USA; edonny@wakehealth.edu; 5 Department of Psychiatry and Behavioral Sciences, University of Minnesota, Minneapolis, MN 55414, USA; hatsu001@umn.edu; 6 Division of Environmental Health Sciences, School of Public Health, University of Minnesota, Minneapolis, MN 55414, USA

**Keywords:** nicotine, low nicotine cigarettes, e-cigarettes, snus, risk perceptions, race, ethnicity, LGBTQ+

## Abstract

Background: As the U.S. Food and Drug Administration considers a low nicotine product standard for cigarettes, it is important to examine how people who smoke, especially individuals from priority populations disproportionately affected by smoking, perceive low nicotine content (LNC) cigarettes and their relative risk perceptions of alternative nicotine delivery system (ANDS) products, including e-cigarettes and snus, and medicinal nicotine. Methods: Data are from Wave 4 (2016–2017) of the adult Population Assessment of Tobacco Use and Health (PATH) Study. We examined respondents’ absolute risk perceptions about nicotine, LNC cigarettes, ANDS products and medicinal nicotine; their relative risk perceptions of LNC cigarettes and ANDS products compared to conventional cigarettes; and their relative risk perceptions of medicinal nicotine compared to ANDS products. Results: The majority of respondents across priority smoking populations indicated snus, e-cigarettes, and LNC cigarettes were ‘about the same’ level of harmfulness or addictiveness as conventional cigarettes. The majority of respondents indicated e-cigarettes to be ‘about the same’ harmfulness as medicinal nicotine. Conclusions: Our study indicates that adults who smoke cigarettes generally have misperceptions about the harms of nicotine and the relative risks of ANDS products and such misperceptions exist regardless of their racial/ethnic identity, sexual orientation, and gender identity.

## 1. Introduction

In 2017, the Food and Drug Administration (FDA) proposed a new regulatory approach for reducing the public health burden of commercial tobacco use in the United States (US) [1]. The nicotine-focused framework developed by FDA regulators relies on the concept that a continuum of harm exists for nicotine and tobacco products—with products such as nicotine replacement therapy representing the least harmful products on this continuum and combusted cigarettes representing the most harmful—and that moving people away from using the most harmful products is essential for improving public health outcomes [2,3]. This framework is founded in the evidence that nicotine, while responsible for the highly addictive nature of commercial tobacco products, is not directly responsible for most tobacco-related disease—rather, it is the other chemicals present in tobacco or tobacco smoke [4]. Cigarettes are the most commonly used commercial tobacco product in the U.S. and pose the greatest threat to consumer health due to the exposure to harmful toxicants via combusted tobacco smoke [5,6,7]. E-cigarettes and snus, two types of alternative nicotine delivery system (ANDS) products, are likely to be less harmful relative to cigarettes because they are not combusted, although the effects of long-term use for some products (e.g., e-cigarettes) are unknown. Finally, medicinal nicotine products, such as nicotine gum and patches, are longstanding FDA-approved pharmacotherapies for smoking cessation with minimal safety concerns for short- or long-term use [8,9]; however, uptake of these products is relatively low with only about 30% of people using medicinal nicotine during cessation attempts [8]. 

As part of this new continuum of harm framework, the FDA proposed implementing a low nicotine product standard for all commercially available cigarettes, which would require cigarettes to have nicotine levels that are minimally or non-addictive [1]. Since nicotine is the primary reinforcing constituent in cigarettes that contributes to establishing and maintaining smoking behavior, drastically reducing the allowable nicotine content could prevent adolescents from becoming dependent on cigarettes and help people who currently smoke cigarettes to quit or cut down [10,11]. Indeed, results from clinical trials consistently report reductions in smoking behavior, toxicant exposure, and nicotine dependence among adult smokers who switched to low nicotine content (LNC) cigarettes [12,13,14,15,16,17,18,19]. Such trials also report minimal evidence of the following potential unintended consequences: compensatory smoking behavior due to decreased nicotine levels, exacerbation of psychiatric symptoms, or increases in alcohol or cannabis use among adults smoking LNC cigarettes [12,16,17,20,21,22,23,24,25,26]. In a simulation study, population estimates indicate that as many as 5 million people will quit smoking within the first year of a low nicotine product standard [27]. However, the public health benefits of a nicotine reduction policy depend on a second component of the nicotine-focused framework: making alternative nicotine products available for people who are unwilling or unable to stop using nicotine. This can be achieved both by supporting innovations in and access to medicinal nicotine as well as via thoughtful regulation of the commercial marketplace of non-medicinal ANDS products [1]. Together, this two-pronged public health approach could result in upwards of 55 million life-years gained over the next 50 years [27]. 

One important consideration for implementing the proposed framework is determining whether the public understands the continuum of harm for commercial nicotine and tobacco products. In general, the relatively minor role of nicotine’s contribution to disease development is not well understood by the public [28]. For example, research on medicinal nicotine highlights the confusion surrounding the harms of nicotine. In prior studies, people frequently reported nicotine gum or patches to be as harmful as smoking cigarettes [29,30]. More recently, a nationally representative survey found that nearly half of respondents incorrectly reported that nicotine causes cancer [31]. Within the context of a low nicotine product standard for cigarettes, this nicotine misperception is problematic because people could misconstrue “reduced nicotine” to mean “reduced harm”. Indeed, Byron et al. reported that nearly half of participants believed LNC cigarettes were less carcinogenic, and this misperception was associated with decreased intentions to quit smoking when asked to predict their behavior if the government mandated that tobacco companies reduce the nicotine in cigarettes [32]. LNC cigarettes are beneficial over conventional cigarettes to the extent that they may promote or facilitate smoking cessation or reductions; however, continued smoking with LNC cigarettes at similar levels as conventional cigarettes would not likely afford users substantial health benefits. Thus, misperceptions about the risks of smoking LNC cigarettes, if they lead to persistence of smoking, could reduce the public health impact of a low nicotine product standard.

As previously stated, e-cigarettes and snus are likely to be less harmful relative to smoking cigarettes, but consumers may not understand the risks of using these products relative to cigarettes or other combusted tobacco products. With regard to e-cigarettes, the risk perceptions literature reports mixed findings. Early research indicated that many groups (e.g., adults, adolescents, people who smoke) perceived e-cigarettes to be less harmful to health relative to cigarettes, which is likely accurate within the context of the tobacco continuum of harm [2,33,34]. However, over time, risk perceptions have shifted towards e-cigarettes being perceived as harmful products [33,34]. For example, Huang et al. found that nearly 65% of respondents in 2017 perceived e-cigarettes to be equally or more harmful than cigarettes, and that this misperception significantly increased over a five-year period [35]. Other studies report substantial misperceptions concerning snus, with people incorrectly believing that using snus is equally or more harmful than smoking cigarettes [36,37,38]. However, the epidemiological data from Sweden indicate the people who use snus have substantially lower risks of developing tobacco-related diseases compared to people who smoke cigarettes [39]. If people who are unable to stop using nicotine believe ANDS products, such as e-cigarettes and snus, are equally or more harmful than cigarettes, then they may not make the switch, thereby reducing the public health impact of the nicotine-focused framework. Importantly, consumer beliefs, such as product risk perceptions, are central tenets of several health behavior theories [40,41], which state that people’s beliefs influence their behaviors. Therefore, targeting risk perceptions to change behavior is a key target in tobacco control research [42,43]. Within the tobacco control literature, there is evidence demonstrating that targeting risk perceptions about tobacco products can change use behaviors, such as reducing initiation and increasing quit attempts [44,45,46]. Therefore, examining risk perceptions of nicotine, LNCs and ANDS products, including e-cigarettes and snus, are critical for understanding how a nicotine-focus framework may affect smoking behavior.

Although the overall smoking prevalence continues to decline in the U.S., cigarette smoking is now more heavily concentrated among underserved and priority populations including racial and ethnic minoritized groups and people identifying as lesbian, gay, bisexual, transgender, queer, and other sexual or gender identities (LGBTQ+) [7]. Factors contributing to the increased smoking prevalence among these groups include systematic racism and/or discrimination, [47,48,49] as well as targeted marketing and advertising campaigns by the tobacco industry [50,51,52,53,54]. Understanding how racial and ethnic minoritized groups and LGBTQ+ people perceive LNC cigarettes and ANDS is important for assessing how the nicotine-focused framework may benefit all populations, including whether the proposed framework may exacerbate existing smoking-related health disparities. Although a few prior LNC and/or ANDS risk perception studies have reported outcomes for people identifying as Black or African American [31,32,55], to our knowledge, this topic has been understudied among people identifying as American Indian or Alaskan Native, Asian, Hispanic, Native Hawaiian or Pacific Islander or more than one race. Further, data concerning risk perceptions among LGBTQ+ populations are also limited: one prior study examining risk perceptions among LGBTQ+ populations was restricted to young adults [55], while another included older people identifying as LGB but did not explicitly examine perceptions among people identifying as transgender [56]. As a result, there are notable gaps in the risk perception literature concerning subgroups within the LGBTQ+ population, with a particular dearth of estimates from nationally representative data sources. Therefore, the primary aims of the current study are (1) to describe the overall trends in risk perceptions of nicotine in general, low nicotine content cigarettes, and two ANDS products (e-cigarettes and snus) using a U.S. representative sample; and (2) to examine the findings among minoritized groups to determine if there are differences in risk perceptions. 

## 2. Materials and Methods

### 2.1. Data Source

Data are from Wave 4 (2016–2017) of the adult Population Assessment of Tobacco Use and Health (PATH) Study Restricted-Use Files. Briefly, PATH is a U.S. representative cohort study of tobacco and nicotine use that started data collection during 2013–2014 (Wave 1). For Wave 1, PATH used a stratified address-based, area-probability sampling design that oversampled tobacco users and African American adults. Survey weights were created that adjusted for the study design and account for nonresponse and over-and under-coverage of certain population groups. The probability sample and weights allow for estimates that are representative of the non-institutionalized, civilian U.S. population. For data collection, field interviewers visited respondents’ homes to conduct interviews using audio computer-assisted self-interviews. At the time of data analysis for the present study, Wave 4 was the most recent wave with public-use data available for analysis. Unique to Wave 4 is a replenishment sample that was combined with the Wave 4 respondents who were from the Wave 1 Cohort to account for loss to follow-up among prior waves. This combined set of Wave 4 participants forms the Wave 4 data and included 33,822 adult respondents. Further details on the PATH methodology can be found elsewhere [57,58]. This study was submitted for review to the University of Minnesota Institutional Review Board but the board determined the study did not constitute human subjects research because of the de-identified nature of the secondary data analysis.

### 2.2. Measures

*Perceptions of harm of cigarettes, non-combustible tobacco products, and nicotine:* The following question was asked separately for each product/product characteristics shown in the brackets: “How harmful do you think [cigarettes/e-cigarettes or other electronic nicotine products/snus/nicotine in nicotine replacement products/nicotine/nicotine in cigarettes/nicotine in e-cigarettes or other electronic nicotine products] is/are to health”. Response options were: “Not at all harmful”, “Slightly harmful”, “Somewhat harmful”, “Very Harmful”, and “Extremely Harmful”. 

*Perceptions of low nicotine cigarettes and non-combustible tobacco products relative to regular cigarettes:* Additionally, we examined the following questions: “In your opinion, do you think cigarettes with lower amounts of nicotine are less harmful, about the same, or more harmful to a person’s health than regular cigarettes?”, “In your opinion, do you think that cigarettes with lower amounts of nicotine are less addictive, about the same, or more addictive than regular cigarettes?”, “Is using [e-cigarettes or other electronic nicotine products/snus] less harmful, about the same, or more harmful than smoking cigarettes?” 

*Perceptions of e-cigarettes relative to nicotine replacement therapy:* Only e-cigarettes were asked about in relation to nicotine replacement therapy with the following question: “Do you think using e-cigarettes or other electronic nicotine products is less harmful, about the same, or more harmful than using nicotine patch, gum, or lozenge?”

*Perceptions of nicotine:* We examined the following two questions on nicotine perceptions “Do you believe nicotine is the main substance in tobacco that makes people want to use tobacco products?”, “Do you believe nicotine is the chemical that causes most of the cancer caused by smoking cigarettes?”. Response options were: “Definitely yes”, “Probably yes”, “Probably not”, “Definitely not”. For all questions, respondents were able to report “I don’t know”. 

*Race and Ethnicity:* To assess the social constructs of race and ethnicity, respondents were asked the following questions: “What is your race?” and “Are you Hispanic, [Latino| Latina], or of Spanish origin?”. For the current study, we classified respondents into six groups, although we recognize that important heterogeneity exists within the groups: (1) American Indian/Alaskan Native (non-Hispanic/Latino/Latina/Spanish origin (HLLS)); (2) Black/African American (non-HLLS); (3) Asian (non-HLLS) which includes Asian Indian, Chinese, Filipino, Japanese, Korean, Vietnamese and Other Asian; (4) Hispanic/Latino/Latina/Spanish origin; (5) Native Hawaiian/Guamanian/Chamorro/Samoan/Other Pacific Islander and those identifying with ‘more than one race’ (non-HLLS); and (6) white (non-HLLS). 

Of note, respondents who identified as Hispanic/ Latino/Latina/Spanish origin were classified into the Hispanic/Latino/Latina/Spanish origin group, regardless of their racial identity. Respondents who identified as American Indian/Alaskan Native in combination with any other race(s) were classified into the American Indian/Alaskan Native group in order to increase the sample size. Prior studies using PATH data have applied a similar classification approach [59,60,61,62]. Finally, due to the small sample sizes for those identifying as Native Hawaiian/Guamanian/Chamorro/Samoan/Other Pacific Islander or ‘more than one race’ (with the exception of American Indian/Alaskan Native), we combined these respondents into one group to increase power.

*Sexual Orientation and Gender Identity:* To assess sexual orientation, respondents were asked the following question: “Do you consider yourself to be straight, lesbian or gay, bisexual, or something else?” To assess gender identity, respondents were asked: “Do you consider yourself to be transgender?” Response options were “Yes” or “No”. Since the question did not explicitly ask if respondents identified as cisgender, gender non-conforming, non-binary, etc., we opted to define those who answered “No” as “nontransgender” rather than make assumptions about their gender identities. 

### 2.3. Analytical Sample

Given the interest in how people who currently smoke perceive low nicotine content cigarettes, ANDS products, and nicotine, data for the present analyses were restricted to people reporting current and established use of cigarettes, which is defined in PATH by the following criteria: respondents who have smoked at least 100 cigarettes in their lifetime, and currently smoke every day or some days. We further restricted the sample to respondents who did not report established use of non-combustible tobacco products (i.e., e-cigarettes or snus) which was defined in PATH by the following criteria: respondents who have ever used (electronic nicotine products/snus), have ever used them fairly regularly, and currently use every day or some days. Experimental users of non-combustible tobacco *products* were therefore included, which is defined in PATH by the following criteria: respondents who have never used (electronic nicotine products/snus) fairly regularly, and currently use every day or some days. 

### 2.4. Statistical Analyses

All response options to perception measures, including “I don’t know”, were examined among the overall sample (i.e., not by race/ethnicity, sexual orientation, or transgender identity) to understand the distribution of perceptions. Sensitivity analyses were conducted among the overall sample by people who smoke daily versus somedays. Due to sample size concerns for comparisons across race/ethnicity group, sexual orientation, and transgender identity, perceptions of harm of cigarettes, non-combustible tobacco products, and nicotine to health were modeled as a continuous measure from 0 (“Not at all harmful”) to 4 (“Extremely harmful”). All other measures were collapsed to a bivariate measure, which compared the misperception versus the accurate perception based on available evidence to date. 

Analyses were conducted using SAS version 9.4. There were minimal missing data (<1–3%) for any given measure. Respondents’ missing measures were excluded from the corresponding analysis. In accordance with the PATH user guidebook [58], analyses were performed with appropriate survey procedures, sampling weights, and variance estimation [63,64] to produce estimates representative of the non-institutionalized, civilian U.S. population at the time of Wave 4 data collection (2016–2017). Data were summarized descriptively via weighted means or proportions and corresponding 95% confidence intervals (CIs). 

For the race/ethnicity comparisons, respondents who identified as white (non-HLS) were used as the reference group for two reasons: (1) to frame the results to highlight implications for minoritized groups that may experience smoking-related disparities resulting from systemic racism and social/economic disadvantage; and (2) white (non-HLS) respondents had the largest sample size and therefore, resulted in the greatest statistical power. Similarly, for the sexual orientation and gender identity comparisons, respondents who identified as straight were used as the reference group for sexual orientation modeling and respondents who identified as non-transgender were used as the reference group for gender identity modeling. Similarly, people identifying as straight and non-transgender were used as the reference groups because members of the LGBTQ+ community experience greater smoking-related disparities attributable to stigma/discrimination and the straight/non-transgender groups had larger sample sizes, which resulted in the greatest statistical power.

## 3. Results

### 3.1. Overall Sample

The overall sample comprised 8340 respondents who reported current and established use of cigarettes but no current and established use of non-combustible tobacco products. Average respondent age was 44.45 years and slightly more than one-half were male (51.88%) and reported that their highest level of educational attainment was high school graduation/GED or less (57.93%).

**Figure 1:**
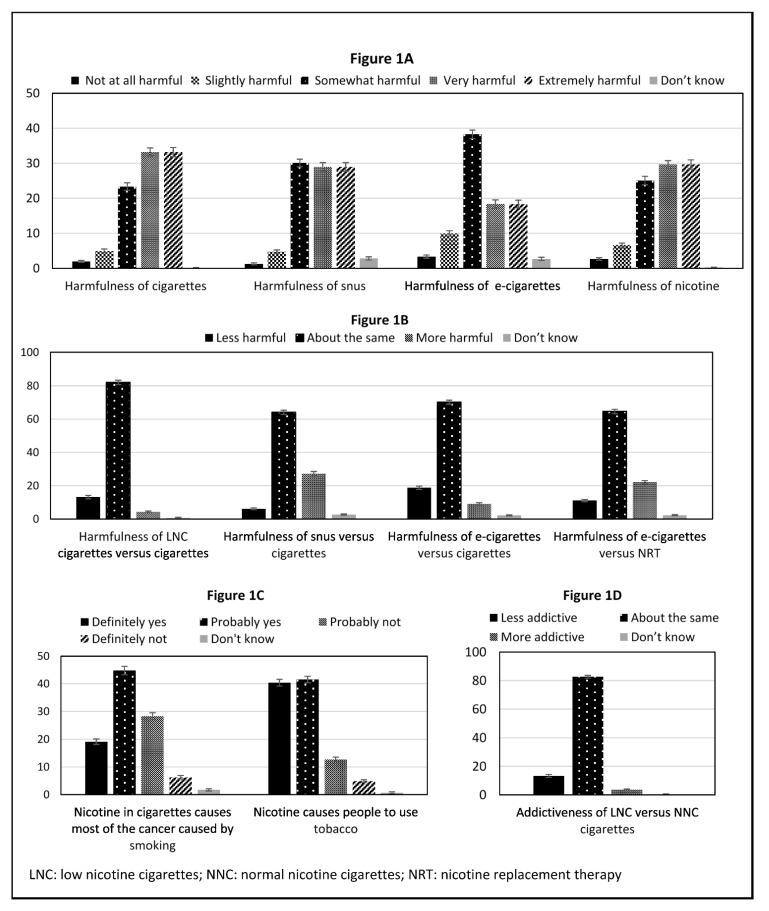
Proportions and 95% confidence intervals for response categories. Persons who currently smoke daily or some days (*n* = 8430). (**A**) displays the perceived harm of products; (**B**) displays the relative risk of products; (**C**) displays health risks of nicotine; (**D**) displays the relative addictiveness of low nicotine cigarettes versus normal nicotine cigarettes.

Distributions of perceptions among the overall sample are shown in Figure 1A–D and Supplementary Table S1. Regarding harmfulness of cigarettes, snus, e-cigarettes, and nicotine, the vast majority of participants perceived these products to be in the range of ‘somewhat harmful’ to ‘extremely harmful’ (Figure 1A). Distribution of responses to harmfulness of e-cigarettes differed the most from that of cigarettes whereby responses of ‘not at all harmful’, ‘slightly harmful’ and ‘somewhat harmful’ were higher for e-cigarettes than cigarettes. Results based on a continuous variable from 0 (not at all harmful) to 4 (extremely harmful) reflect these distributions, whereby the mean response varied slightly from 2.49 for e-cigarettes to 2.94 for cigarettes (Supplementary Table S1). Regarding harmfulness relative to cigarettes (Figure 1B), the majority of respondents indicated LNC cigarettes, snus, and e-cigarettes to be ‘about the same’ harmfulness as cigarettes. Relative to NRT, the majority of respondents indicated e-cigarettes to be ‘about the same’ harmfulness as NRT and the distribution of responses nearly mirrored the distribution of responses when harmfulness of e-cigarettes was relative to cigarettes. These relationships are likely explained by Figure 1C, which shows that nearly two-thirds (63.83%) of respondents reported “definitely yes” or “probably yes” to the statement ‘*nicotine in cigarettes causes most of the cancer caused by smoking*’. Finally, despite the vast majority (81.89%) of respondents reporting “definitely yes” or “probably yes” to the statement ‘*nicotine causes people to use tobacco*’ (Figure 1C), most respondents reported that LNC cigarettes had “about the same” level of addictiveness as normal nicotine content (NNC) cigarettes (Figure 1D).

Distributions of perceptions among respondents who smoke daily (i.e., removing respondents who smoked some days, *n* = 1785) are shown in Supplementary Figure S1A–D and Supplementary Table S1. Results were similar to those when including respondents who smoke some days. For these reasons, analyses comparing across race/ethnicity, sexual orientation, and gender identity combine respondents who currently smoke cigarettes daily and respondents who currently smoke somedays. 

### 3.2. Race/Ethnicity Group Comparisons

**Figure 2:**
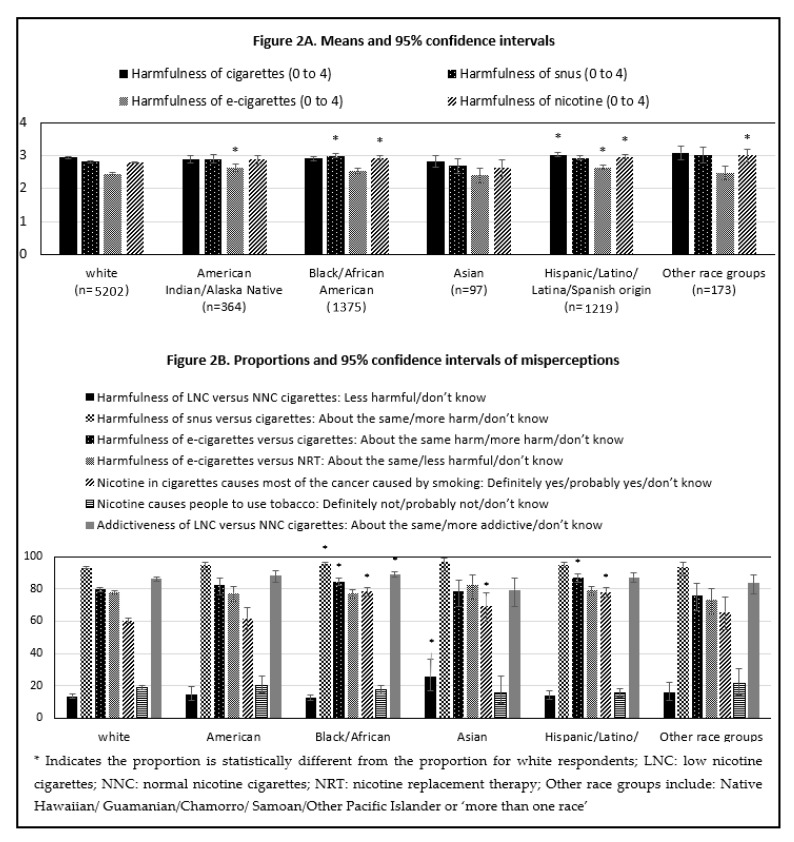
Means or proportions and 95% confidence intervals for response categories. Persons who currently smoke daily or some days by race/ethnicity. (**A**) displays the perceived harm of products by racial and ethnic groups; (**B**) displays the proportion of respondents endorsing a misperception about nicotine or the relative risks of products by racial and ethnic group.

As shown in Figure 2A and Table 1, patterns in mean responses to harmfulness of cigarettes, snus, e-cigarettes, and nicotine were similar across the multiple race and ethnicity groups with a few notable differences. The perceived harmfulness of e-cigarettes was significantly higher among American Indian/Alaskan Native respondents relative to white respondents. The perceived harmfulness of snus and nicotine was significantly higher among Black/African American respondents relative to white respondents. Similar observations were seen for Hispanic/Latino/Latina/Spanish origin respondents, but this is also within the context of this group viewing cigarettes as more harmful than white respondents. 

Figure 2B and Table 1 show proportions of misperceptions which include the response ‘don’t know’ by race/ethnicity. Between 12.38 and 25.53% of respondents across race and ethnicity groups reported a misperception to the statement on the harmfulness of LNC versus NNC cigarettes and the proportion was significantly higher for Asian respondents (25.53%) when compared with white respondents (12.38%). Misperceptions regarding harmfulness of snus and e-cigarettes versus cigarettes were represented among the vast majority (>75%) of respondents across race/ethnicity groups. A significantly greater proportion of Black/African American versus white respondents misperceived the harmfulness of snus and e-cigarettes relative to cigarettes. A significantly greater proportion of Hispanic/Latino/Latina/Spanish origin versus white respondents misperceived the harmfulness of e-cigarettes compared to cigarettes. The majority of respondents (>60%) across race/ethnicity groups indicated a misperception to the statement ‘*nicotine in cigarettes causes most of the cancer caused by smoking*’ and the proportion of respondents with the misperception was significantly higher among Black/African American (78.41%), Asian (69.53%) and Hispanic/Latino/Latina/Spanish origin (77.99%) versus white respondents (60.18%). Finally, the vast majority of respondents had a misperception about the addictiveness of LNC versus NNC cigarettes, with respondents reporting that LNC cigarettes are ‘about the same’ addictiveness as NNC cigarettes. 

### 3.3. Sexual Orientation and Gender Identity Comparisons

**Figure 3:**
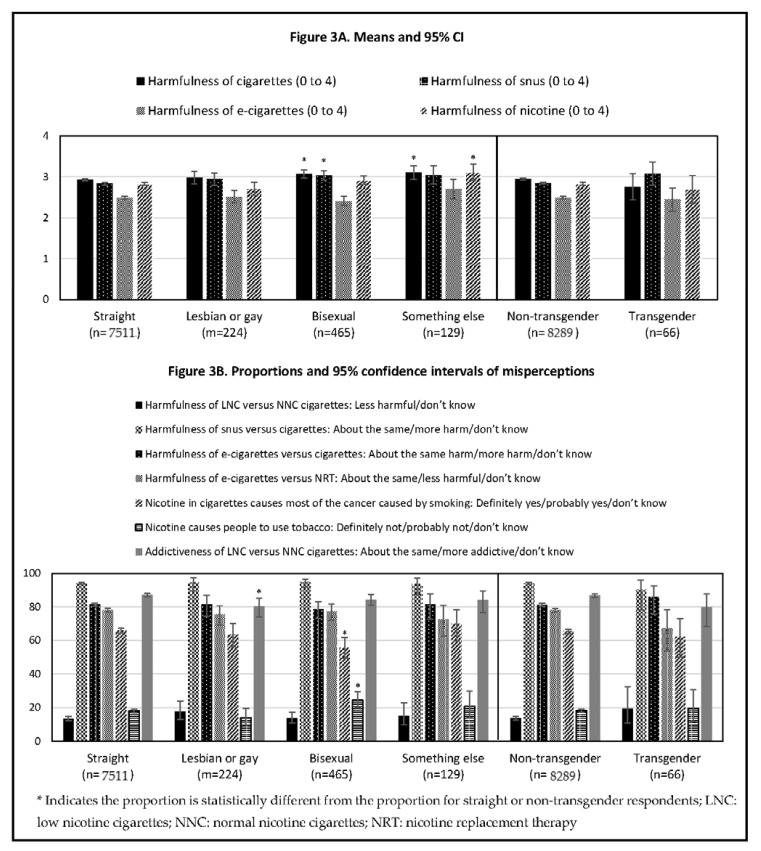
Means or proportions and 95% confidence intervals for response categories. Persons who currently smoke daily or some days by sexual orientation and gender identity. (**A**) displays the perceived harm of products by sexual orientation and gender identity; (**B**) displays the proportion of respondents endorsing a misperception about nicotine or the relative risks of products by sexual orientation and gender identity.

As shown in Figure 3A and Table 2 and Table 3, patterns in mean responses to the harmfulness of cigarettes, snus, e-cigarettes, and nicotine were similar across sexual orientation and gender identity groups and reflect the overall population findings. However, there were a few significant differences. Perceived harmfulness of cigarettes and snus was higher for respondents who were bisexual versus straight respondents. Perceived harmfulness of cigarettes and nicotine in general was higher for respondents who were reported their sexual orientation as ‘something else’ versus straight.

Overall, as shown in Figure 3B and Table 3, misperceptions were similar across sexual orientation and gender identity groups but with a few significant differences. The proportion who had a misperception on the addictiveness of LNC versus NNC cigarettes was lower in respondents who identified as lesbian or gay versus straight. The proportion who had a misperception to the statement ‘*nicotine in cigarettes causes most of the cancer caused by smoking*’ was lower in respondents who were bisexual compared to straight, while the proportion of respondents who had a misperception to the statement ‘*nicotine causes people to use tobacco*’ was higher in respondents who were bisexual compared to straight. 

## 4. Discussion

Our results indicate that adults in the U.S. who smoke cigarettes generally have misperceptions about the harms of nicotine and the relative risks of ANDS products, which is consistent with findings from prior research [29,32,65]. For most of the risk perception questions we examined from the PATH survey, the majority of respondents, regardless of their racial/ethnic identity, sexual orientation, and gender identity, reported misperceptions. This is concerning because the FDA’s nicotine-focused framework relies on people who are unable or unwilling to quit nicotine to differentiate the harms of the different products. The most harmful delivery system of nicotine is the cigarette and other combusted products because the user is exposed to toxicants in the tobacco smoke that cause cancer and other smoking-related illnesses [6]. However, based on our findings from the relative risk questions, most people who smoke cigarettes do not differentiate between the harms of using cigarettes and the harms of using potentially reduced risk ANDS products, indicating that they do not understand the tobacco continuum of harm. For example, when comparing snus to cigarettes, over 90% of respondents indicated the non-combusted product was equally or more harmful than cigarettes. This finding is especially interesting in light of the fact that the FDA’s first approved ‘modified risk tobacco product’ application from Swedish Match was for the following snus relative risk claim: “*Using General Snus instead of cigarettes puts you at lower risk of mouth cancer, heart disease, lung cancer, stroke, emphysema, and chronic bronchitis*” [66]. A recent study found that when people who smoke were exposed to snus advertisements that included modified risk claims, the majority of respondents accurately reported lower relative risk perceptions for snus compared to cigarettes but also understood that snus is not completely harmless [67]. Thus, health communication campaigns explaining the tobacco continuum of harm, including the potential risks and benefits of using non-combusted products in place of cigarettes, will be critical when implementing a nicotine-focused framework.

Since risk perceptions contribute to behavior [40,41], examining perceptions by race/ethnicity, sexual orientation, and gender identity can help inform how these populations may respond to a low nicotine product standard in cigarettes. Moreover, early identification of misperceptions can provide an opportunity to prepare tailored messages prior to and concurrently with the implementation of a low nicotine product standard for cigarettes. In the current study, we found that the risk perception ratings across the populations studied generally aligned with the overall results and suggest that most people are not well-informed about the harms of nicotine. For example, nearly three-quarters of respondents indicated that the nicotine in medicinal nicotine products is somewhat to extremely harmful, which may partially explain why so few people use NRT during quit attempts [8]. As such, some people who want to quit smoking may benefit from other cessation approaches, such as mHealth applications or other pharmacotherapies (e.g., varenicline), to support their quit attempts, if efforts to correct misperceptions about medicinal nicotine risks are not successful. Overall, our results suggest that the majority of adults who smoke cigarettes across all priority populations could benefit from both *nicotine-specific* education, i.e., explaining the risks of addiction and correcting misperceptions about the health risks of nicotine, and *product-specific* education, i.e., explaining the relative risks across products containing nicotine. 

That being said, there were a few notable differences in risk perceptions across the groups. First, a significantly greater proportion of respondents identifying as Black/African American, Asian and Hispanic/Latino/Latina/Spanish origin relative to white respondents incorrectly attributed nicotine as the *‘chemical that causes most of the cancer caused by smoking cigarettes*’, although the majority of respondents also indicated this misperception. Respondents who identified as American Indian/Alaskan Native and Hispanic/Latino/Latina/Spanish origin had significantly higher e-cigarette risk ratings relative to respondents identifying as white. Respondents identifying as Black/African American, Hispanic/Latino/Latina/Spanish origin, bisexual, or another sexual orientation reported higher risk perceptions ratings for snus relative to respondents identifying as white or straight. Together, these findings could be interpreted to suggest that some people from minoritized groups may be less willing to switch to ANDS products if the FDA implements a low nicotine product standard for cigarettes. If these individuals instead continue to smoke cigarettes (i.e., LNC cigarettes), then the full benefits of a nicotine reduction policy for these minoritized groups may be attenuated and, as a result, widen existing smoking disparities. However, another interpretation is that more people from minoritized groups would quit tobacco altogether—as the robust VLNC literature indicates that use of VLNC cigarettes increases the likelihood of making a quit attempt or achieving abstinence [12,13,18,68,69,70,71,72]—versus switching to ANDS products. These findings merit further exploration. To date, little research is available examining the impact of a low nicotine product standard for cigarettes on smoking and quitting behavior among most of the minoritized groups examined in the current study.

Developing and implementing targeted communication interventions for different racial/ethnic groups as well as sexual orientation and gender identity would allow public health officials to more effectively communicate with these subgroups based on their unique values, perceptions of tobacco products, and causes of tobacco use (e.g., systemic racism). Targeting messages to specific populations enhances its effectiveness, because messages are most effective when they are perceived as relevant to a population [73,74]. One successful example of targeted messaging is *The Real Cost* national campaign, aimed at preventing youth from initiating smoking [45,46,75]. However, we are unaware of any existing messaging for many of the minoritized groups examined in this study, with a few exceptions [76,77,78]. Thus, future research should explore optimal ways to communicate to these groups about a low nicotine product standard for cigarettes and the tobacco continuum of harm. 

Interestingly, the only question for which the majority of respondents answered correctly was that ‘*nicotine causes people to use tobacco*’. In contrast, across the different populations studied, between 79 and 89% of respondents reported the misperception that LNC cigarettes had ‘about the same’ level of addictiveness as NNC cigarettes. Our findings highlight an important disconnect in understanding of LNC cigarettes with regard to the measures used in the present study. Potential reasons for this disconnect may include that participants interpreted the question on LNC cigarettes as referring to “light” or “ultra-light” cigarettes, which were falsely promoted by the tobacco industry as safer cigarettes [78]. Another possible reason for the observed disconnect is that the nicotine level of LNC cigarettes was never quantified in the survey measure. Recent studies have shown that stating “95% of the nicotine would be removed”, which reflects levels of nicotine in LNC cigarettes versus conventional cigarettes, results in more accurate perceptions of the addictiveness of LNC cigarettes [79]. Another observed misperception is that over 60% of respondents indicated that it is the nicotine in cigarettes causing cancer, which is consistent with prior studies including one among U.S. physicians [31,32,80]. Again, such findings point to the need for educating the public about the harms of nicotine and LNC cigarettes prior to implementing a low nicotine product standard for cigarettes. As shown in prior research by Villanti and colleagues, brief communication interventions can be effective in correcting misperceptions of LNC cigarettes, ANDS products, and medicinal nicotine [28]. 

Our findings must be considered within the context of a few limitations. First, Wave 4 recruitment occurred in 2016–2017, so perceptions of alternative products may have changed over time. Second, new nicotine products, such as heated tobacco products and oral nicotine pouches, are now available and should be examined within the context of the nicotine-focused framework, especially since IQOS, a heated tobacco product, has received approval by the FDA to be marketed as a modified risk tobacco product [81]. Future research may want to examine perceptions of IQOS’ reduced exposure claims relative to LNC cigarettes and other ANDS products. Third, as previously described, the wording of the question assessing the relative addictiveness of low nicotine cigarettes compared to conventional cigarettes was vague, which may have contributed to misperceptions. Fourth, we opted to focus on snus specifically rather than all smokeless tobacco products because of the robust epidemiological literature supporting reduced health risks compared to smoking [32]. Studies examining absolute and relative risk perceptions of other smokeless tobacco products may also be important for understanding how the public perceives the tobacco continuum of harm. Fifth, this study did not examine adolescent risk perceptions, a population for which e-cigarettes are the most frequently used tobacco product [82]. One challenge for communicating risks of using ANDS relative to smoking cigarettes will be to do so in a manner that encourages product switching among adults who are unwilling or unable to stop smoking but that does not encourage adolescents to experiment with the products because they may misinterpret reduced harm to mean no harm. More communication research is needed that balances adolescent prevention and adult harm reduction messaging. Finally, we want to reiterate that race and ethnicity are social and not biological constructs. Grouping respondents into mutually exclusive categories increased our sample sizes and subsequently our statistical power to identify differences across the groups. However, this approach limited our ability to examine the important within-group differences that exist across minoritized groups, such as American Indian/Alaskan Native and Asian populations and the LGBTQ+ community. This study is a first step in understanding the risk perceptions of LNC cigarettes and ANDS products among understudied, minoritized groups; however, more research is needed to understand the nuances within groups in order to inform future health communication campaigns.

## 5. Conclusions

The majority of adults who smoke cigarettes reported misperceptions about nicotine and nicotine-containing products with relatively consistent findings across different priority smoking populations. Thus, if the FDA intends to move forward with implementing a nicotine-focused framework to improve public health, then time and resources are needed to educate the public about the tobacco continuum of harm and to correct misperceptions about nicotine, low nicotine content cigarettes, ANDS products, including e-cigarettes and snus, and medicinal nicotine. Additional targeted messages for racial, ethnic and LGBTQ+ groups may also be beneficial for ensuring that all smoking populations benefit from a nicotine-focused framework.

## Figures and Tables

**Table 1:** Weighted mean values or weighted proportions and corresponding 95% confidence intervals; PATH Wave 4 adults; Current established daily or someday smokers.

	White (*n* = 5202)			American Indian/Alaskan Native (*n* = 364)			Black/African American (*n* = 1375)			Asian (*n* = 97)			Hispanic/Latino/Latina/ Spanish Origin (*n* = 1219)			Native Hawaiian/ Guamanian/Chamorro/Samoan/Other Pacific Islander or ‘More Than One Race’ (n = 173)			Global *p*-Value
	**% or µ**	**LCL**	**UCL**	**% or µ**	**LCL**	**UCL**	**% or µ**	**LCL**	**UCL**	**% or µ**	**LCL**	**UCL**	**% or µ**	**LCL**	**UCL**	**% or µ**	**LCL**	**UCL**	
**Age (years), µ**	45.18	44.58	45.77	45.78	43.88	47.68	45.38	44.49	46.27	41.21 *	37.64	44.78	40.80 ***	39.90	41.69	34.97 ***	32.27	37.67	<0.0001
**Gender, %**																			<0.0001
Male	49.13	47.44	50.83	49.84	44.23	55.46	54.00 *	49.94	58.00	73.33 ***	61.47	82.57	60.35 ***	56.34	64.23	55.63	45.82	65.02	
Female	50.87	49.17	52.56	50.16	44.54	55.77	46.00	42.00	50.06	26.67	17.43	38.53	39.65	35.77	43.66	44.37	34.98	54.18	
**Educational attainment, %**																			<0.0001
HS graduate/GED or less	55.97	54.19	57.74	54.09	47.73	60.31	63.41 ***	60.46	66.26	21.43 ***	13.69	31.91	68.27 ***	64.71	71.63	49.60	40.65	58.58	
At least some college	44.03	42.26	45.81	45.91	39.69	52.27	36.59	33.74	39.53	78.57	68.09	86.31	31.73	28.37	35.29	50.40	41.42	59.35	
**Smoking status, %**																			<0.0001
Daily	83.40	81.90	84.79	81.71	76.01	86.29	73.32 ***	70.47	75.99	63.67 ***	52.27	73.72	61.08 ***	57.42	64.62	78.17	70.86	84.05	
Somedays	16.60	15.21	18.10	18.29	13.71	23.99	26.68	24.01	29.53	36.33	26.28	47.73	38.92	35.38	42.58	21.83	15.95	29.14	
**Harmfulness of cigarettes (0–4), µ**	2.94	2.91	2.97	2.88	2.76	3.00	2.91	2.85	2.96	2.82	2.65	3.00	3.03 **	2.97	3.09	3.08	2.87	3.28	0.0215
**Harmfulness of snus (0 to 4), µ**	2.81	2.78	2.84	2.90	2.78	3.02	2.98 ***	2.92	3.05	2.69	2.47	2.91	2.93 **	2.87	2.99	3.01	2.77	3.25	<0.0001
**Harmfulness of e-cigarettes (0 to 4), µ**	2.45	2.41	2.48	2.63 **	2.51	2.75	2.53	2.46	2.60	2.40	2.18	2.61	2.64 ***	2.58	2.70	2.47	2.27	2.67	<0.0001
**Harmfulness of nicotine (0 to 4), µ**	2.79	2.76	2.82	2.88	2.75	3.01	2.93 ***	2.86	3.00	2.62	2.37	2.86	2.96 ***	2.89	3.02	3.00 *	2.80	3.19	<0.0001
**Harmfulness of nicotine in cigarettes (0 to 4), µ**	2.70	2.67	2.73	2.73	2.60	2.85	2.90 ***	2.85	2.96	2.58	2.36	2.80	2.96 ***	2.89	3.02	2.94 *	2.74	3.14	<0.0001
**Harmfulness of nicotine in e-cigarettes (0 to 4), µ**	2.36	2.33	2.40	2.51 *	2.39	2.63	2.46 *	2.39	2.53	2.25	2.05	2.45	2.64 ***	2.57	2.70	2.39	2.19	2.59	<0.0001
**Harmfulness of nicotine in NRT** **(0 to 4), µ**	2.08	2.04	2.12	2.16	2.05	2.27	2.36 ***	2.29	2.43	2.14	1.87	2.40	2.38 ***	2.31	2.45	2.36	2.12	2.60	<0.0001
**Harmfulness of LNC relative to NNC**																			
*Misperception*: Less harmful/don’t know	13.52	12.18	15.00	14.59	10.64	19.68	12.38	10.67	14.33	25.53 **	16.94	36.55	13.93	11.37	16.97	15.58	10.62	22.28	0.0073
**Addictiveness of LNC relative to NNC**																			
*Misperception*: About the same/more addictive/don’t know	86.39	85.05	87.63	88.14	84.14	91.24	88.88 *	87.06	90.47	79.30	68.81	86.93	87.33	84.42	89.77	83.53	76.89	88.55	0.0398
**Nicotine in cigarettes causes most of the cancer caused by smoking**																			
*Misperception*: Definitely yes/probably yes/don’t know	60.18	58.52	61.82	61.88	54.62	68.64	78.41 ***	75.48	81.08	69.53 *	59.78	77.80	77.99 ***	74.79	80.88	65.56	54.80	74.93	<0.0001
**Nicotine causes people to use tobacco**																			
*Misperception*: Definitely not/probably not/don’t know	18.76	17.49	20.10	20.17	15.50	25.81	17.32	15.05	19.86	15.80	8.99	26.28	15.42	13.14	18.01	21.50	14.45	30.75	0.2309
**Harmfulness of snus versus cigarettes**																			
*Misperception:* Same harm/more harm/don’t know	93.19	92.48	93.85	94.93	91.94	96.84	95.80 ***	94.55	96.78	96.72	90.99	98.85	94.87	92.97	96.28	93.65	88.25	96.67	0.0027
**Harmfulness of e-cigarettes versus cigarettes**																			
*Misperception:* Same harm/more harm/don’t know	79.57	78.29	80.80	82.17	76.18	86.92	84.51 **	81.95	86.77	78.45	69.27	85.47	87.17 ***	84.33	89.56	75.87	66.50	83.28	<0.0001
**Harmfulness of e-cigarettes versus NRT**																			
*Misperception:* About the same/less harmful/don’t know	77.89	76.71	79.02	77.29	72.51	81.46	76.99	74.27	79.50	82.49	73.74	88.78	78.93	76.00	81.60	73.15	64.69	80.21	0.5190

* *p*-value < 0.05; ** *p*-value < 0.01; *** *p*-value < 0.001; LCL: lower 95% confidence interval; UCL: upper 95% confidence interval; HS: High school; LNC: Low nicotine cigarettes; NNC: Normal nicotine cigarettes; NRT: nicotine replacement therapy.

**Table 2:** Weighted mean values or weighted proportions and corresponding 95% confidence intervals; PATH Wave 4 adults; Current established daily or someday smokers who are not established users of non-combustible products by sexual orientation.

	Straight (*n* = 7511)			Lesbian or Gay (*n* = 224)			Bisexual (*n* = 465)			Something Else (*n* = 129)			Global *p*-Value
	% or µ	LCL	UCL	% or µ	LCL	UCL	% or µ	LCL	UCL	% or µ	LCL	UCL	
**Age (years), µ**	45.03	44.55	45.51	39.90 ***	37.39	42.41	34.38 ***	33.28	35.49	39.76 **	36.54	42.99	<0.0001
**Gender, %**													<0.0001
Male	53.17	51.68	54.65	55.84	48.84	62.62	23.31 ***	18.51	28.91	46.82	37.27	56.60	
Female	46.83	45.35	48.32	44.16	37.38	51.16	76.69	71.09	81.49	53.18	43.40	62.73	
**Educational attainment, %**													0.0098
HS graduate/GED or less	58.26	56.69	59.81	46.23 **	36.75	55.99	54.08	49.26	58.82	62.67 *	52.73	71.64	
At least some college	41.74	40.19	43.31	53.77	44.01	63.25	45.92	41.18	50.74	37.33	28.36	47.27	
**Smoking status, %**													0.1189
Daily	78.73	77.48	79.94	81.24	75.41	85.94	74.82	69.81	79.25	71.43	59.78	80.80	
Someday	21.27	20.06	22.52	18.76	14.06	24.59	25.18	20.75	30.19	28.57	19.20	40.22	
**Harmfulness of cigarettes (0 to 4), µ**	2.93	2.91	2.96	2.98	2.83	3.14	3.07 **	2.97	3.18	3.11 *	2.95	3.27	0.0027
**Harmfulness of snus (0 to 4), µ**	2.84	2.81	2.86	2.95	2.79	3.10	3.04 ***	2.92	3.15	3.05	2.83	3.27	0.0007
**Harmfulness of e-cigarettes (0 to 4), µ**	2.49	2.46	2.52	2.51	2.37	2.66	2.41	2.30	2.52	2.71	2.47	2.95	0.1118
**Harmfulness of nicotine (0 to 4), µ**	2.83	2.80	2.86	2.72	2.55	2.88	2.92	2.82	3.03	3.12 **	2.92	3.32	0.0036
**Harmfulness of nicotine in cigarettes (0 to 4),** **µ**	2.76	2.73	2.79	2.73	2.58	2.89	2.82	2.71	2.94	2.96	2.77	3.14	0.0976
**Harmfulness of nicotine in e-cigarettes (0 to 4),** **µ**	2.41	2.39	2.44	2.48	2.31	2.65	2.40	2.27	2.52	2.71	2.49	2.93	0.0584
**Harmfulness of nicotine in NRT (0 to 4),** **µ**	2.16	2.13	2.19	2.15	1.97	2.33	2.24	2.11	2.37	2.46	2.23	2.68	0.0677
**Harmfulness of LNC versus NNC cigarettes, %**													0.3211
*Misperception*: Less harmful/don’t know	13.45	12.31	14.67	17.65	12.77	23.90	13.57	10.67	17.11	15.15	9.75	22.78	
**Addictiveness of LNC versus NNC cigarettes, %**													0.0059
*Misperception*: About the same/more addictive/don’t know	87.07	86.01	88.06	80.25 **	74.08	85.24	84.39	80.81	87.41	84.06	76.51	89.51	
**Nicotine in cigarettes causes most of the cancer caused by smoking, %**													0.0026
*Misperception*: Definitely yes/probably yes/don’t know	65.99	64.53	67.41	63.55	56.35	70.19	55.60 **	49.53	61.51	69.70	59.54	78.24	
**Nicotine causes people to use tobacco, %**													0.0089
*Misperception*: Definitely not/probably not/don’t know	17.84	16.81	18.93	13.94	9.80	19.46	24.28 **	19.79	29.41	20.77	13.91	29.85	
**Harmfulness of snus versus cigarettes, %**													0.9356
*Misperception:* Same harm/more harm/don’t know	93.87	93.27	94.42	94.50	89.36	97.23	94.65	91.78	96.55	93.67	87.12	97.01	
**Harmfulness of e-cigarettes versus cigarettes, %**													0.7235
*Misperception:* Same harm/more harm/don’t know	81.30	80.26	82.29	81.42	74.33	86.89	78.56	73.17	83.12	81.35	72.55	87.80	
**Harmfulness of e-cigarettes versus NRT, %**													0.5223
*Misperception:* About the same/less harmful/don’t know	78.01	76.92	79.07	75.44	69.32	80.67	77.18	72.00	81.65	72.55	62.36	80.84	

* *p*-value < 0.05 as compared to NH Whites; ** *p*-value < 0.01 as compared to NH Whites; *** *p*-value < 0.001 as compared to NH Whites; LCL: lower 95% confidence interval; UCL: upper 95% confidence interval; HS: High school; LNC: Low nicotine cigarettes; NNC: Normal nicotine cigarettes; NRT: nicotine replacement therapy.

**Table 3:** Weighted mean values or weighted proportions and corresponding 95% confidence intervals; PATH Wave 4 adults; Current established daily or someday smokers who are not established users of non-combustible products by transgender identity.

	Non-Transgender (*n* = 8289)			Transgender (*n* = 66)			*p*-Value
	% or µ	LCL	UCL	% or µ	LCL	UCL	
**Age (years), µ**	44.42	43.95	44.88	40.57	36.39	44.76	0.0769
**Gender, %**							0.0813
Male	51.69	50.32	53.06	64.38	50.14	76.46	
Female	48.31	46.94	49.68	35.62	23.54	49.86	
**Educational attainment, % **							0.0130
HS graduate/GED or less	57.73	56.20	59.24	72.94	61.18	82.17	
At least some college	42.27	40.76	43.80	27.06	17.83	38.82	
**Smoking status, %**							0.5351
Daily	78.47	77.26	79.63	81.98	69.74	89.99	
Somedays	21.53	20.37	22.74	18.02	10.01	30.26	
**Harmfulness of cigarettes (0 to 4), µ**	2.94	2.92	2.97	2.76	2.44	3.08	0.2568
**Harmfulness of snus (0 to 4), µ**	2.85	2.83	2.88	3.08	2.80	3.36	0.1114
**Harmfulness of e-cigarettes (0 to 4), µ**	2.49	2.47	2.52	2.45	2.16	2.74	0.7713
**Harmfulness of nicotine (0 to 4), µ**	2.84	2.81	2.87	2.70	2.36	3.04	0.4443
**Harmfulness of nicotine in cigarettes (0 to 4), µ**	2.77	2.74	2.79	2.59	2.30	2.89	0.2457
**Harmfulness of nicotine in e-cigarettes (0 to 4), µ**	2.42	2.39	2.45	2.36	2.08	2.64	0.6753
**Harmfulness of nicotine in NRT (0 to 4), µ**	2.17	2.13	2.20	2.25	1.92	2.57	0.6211
**Harmfulness of LNC versus NNC cigarettes, % **							
*Misperception*: Less harmful/don’t know	13.54	12.45	14.70	19.43	10.82	32.42	0.2266
**Addictiveness of LNC versus NNC cigarettes, % **							
*Misperception*: About the same/more addictive/don’t know	86.79	85.75	87.77	79.67	68.46	87.62	0.0598
**Nicotine in cigarettes causes most of the cancer caused by smoking, %**							
*Misperception*: Definitely yes/probably yes/don’t know	65.52	64.19	66.83	62.24	50.08	73.03	0.5554
**Nicotine causes people to use tobacco, %**							
*Misperception*: Definitely not/probably not/don’t know	18.05	17.05	19.11	19.43	11.64	30.64	0.7639
**Harmfulness of snus versus cigarettes, % **							
*Misperception:* Same harm/more harm/don’t know	93.94	93.38	94.46	90.20	78.16	95.95	0.3119
**Harmfulness of e-cigarettes versus cigarettes, % **							
*Misperception:* Same harm/more harm/don’t know	81.21	80.24	82.15	86.04	75.60	92.46	0.2713
**Harmfulness of e-cigarettes versus NRT, % **							
*Misperception:* About the same/less harmful/don’t know	77.93	76.91	78.92	67.25	53.67	78.45	0.0690

LCL: lower 95% confidence interval; UCL: upper 95% confidence interval; HS: High school; LNC: Low nicotine cigarettes; NNC: Normal nicotine cigarettes; NRT: nicotine replacement therapy.

## Data Availability

All analyses were performed in the Inter-university Consortium for Political and Social Research’s Virtual Data Enclave (VDE) using the PATH Restricted Use Files [55]. Details on how to access these data are found here: https://www.icpsr.umich.edu/web/NAHDAP/studies/36231, accessed 15 January–15 March 2021.

## Supplementary Materials

The following are available online at https://www.mdpi.com/article/10.3390/ijerph18105311/s1, Figure S1: Proportions and 95% confidence intervals for all response categories, Table S1: Weighted mean values or weighted proportions and corresponding 95% confidence intervals.
