# Supplementary material for: Risk Perceptions of Low Nicotine Cigarettes and Alternative Nicotine Products across Priority Smoking Populations
Source: Int J Environ Res Public Health. 2021 May 17;18(10):5311. doi: 10.3390/ijerph18105311 (PMC8156883; doi:10.3390/ijerph18105311)
Supplement: Supplementary file 1 [file ijerph-18-05311-s001.zip › ijerph-1211505-supplementary.pdf]

Supplementary Figure S1. Proportions and 95% confidence intervals for all response categories
Persons who currently smoke daily (n=6645)

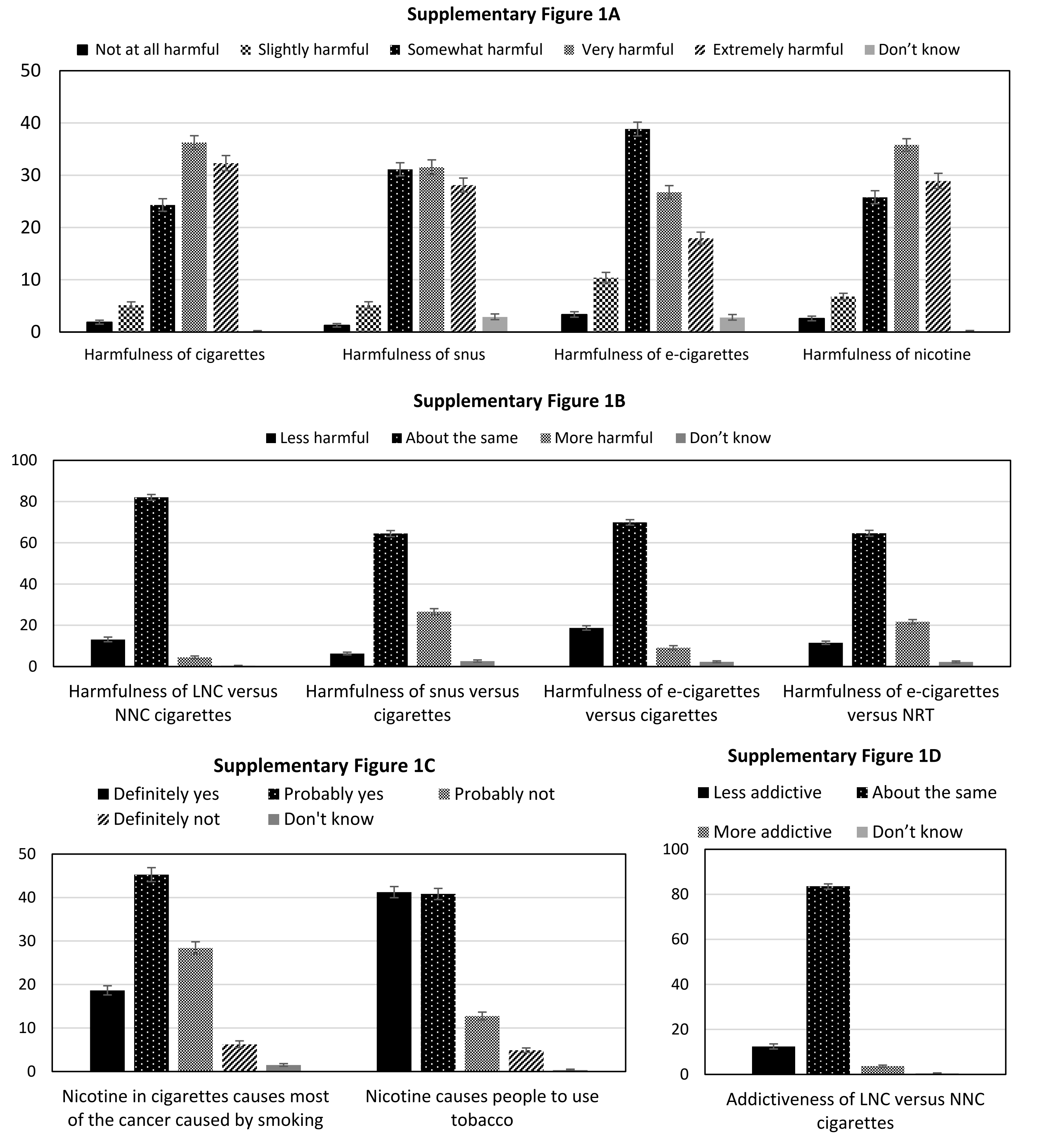

LNC: low nicotine cigarettes; NNC: normal nicotine cigarettes; NRT: nicotine replacement therapy

Supplementary Figure S1. Figure S1A displays the perceived harm of products; Figure S1B displays the relative risk of products; Figure S1C displays health risks of nicotine; and FigureS 1D displays the relative addictiveness of low nicotine cigarettes versus normal nicotine cigarettes.

Supplementary Table S1. Weighted mean values or weighted proportions and corresponding 95% confidence intervals; PATH Wave 4 adults; Current established daily or someday smokers who are not established users of non-combustible products

	**All smokers (n=8430)**			**Daily smokers (n=6645)**			**Someday smokers (n=1785)**			
	**% or μ**	**LCL**	**UCL**	**% or μ**	**LCL**	**UCL**	**% or μ**	**LCL**	**UCL**	*P-value*[1]
**Age (years), μ**	44.45	44.00	44.90	45.21	44.79	45.62	41.67	40.59	42.76	**<.0001**
**Gender, %**										0.8028
Male	51.88	50.52	53.24	51.80	50.25	53.33	52.20	49.38	55.00	
Female	48.12	46.76	49.48	48.20	46.67	49.75	47.80	45.00	50.62	
**Educational attainment, %**										**<.0001**
HS graduate/GED or less	57.93	56.44	59.42	60.77	59.21	62.32	47.57	44.38	50.79	
At least some college	42.07	40.58	43.56	39.23	37.68	40.79	52.43	49.21	55.62	
**Harmfulness of cigarettes, %**										
Not at all harmful	1.94	1.65	2.28	1.86	1.51	2.28	2.25	1.54	3.28	
Slightly harmful	4.93	4.39	5.54	5.13	4.56	5.77	4.20	3.16	5.57	
Somewhat harmful	23.32	22.25	24.42	24.30	23.12	25.51	19.75	17.50	22.21	
Very harmful	36.49	35.31	37.69	36.25	34.95	37.57	37.37	34.68	40.14	
Extremely harmful	33.17	31.89	34.49	32.31	30.89	33.77	36.31	33.54	39.18	
Don't know	0.14	0.08	0.25	0.15	0.08	0.27	0.12	0.03	0.41	
**Continuous measure (0 to 4), μ**	2.94	2.92	2.97	2.92	2.89	2.95	3.01	2.96	3.07	**0.0034**
**Harmfulness of snus, %**										
Not at all harmful	1.26	1.01	1.55	1.25	0.97	1.62	1.28	0.84	1.93	
Slightly harmful	4.69	4.18	5.27	5.13	4.53	5.80	3.11	2.32	4.16	
Somewhat harmful	30.09	29.02	31.18	31.13	29.90	32.39	26.26	23.64	29.07	
Very harmful	32.25	30.97	33.55	31.53	30.14	32.95	34.89	32.06	37.82	
Extremely harmful	28.91	27.65	30.21	28.10	26.77	29.47	31.89	28.73	35.22	
Don't know	2.81	2.38	3.31	2.87	2.37	3.46	2.58	1.81	3.67	
**Continuous measure (0 to 4), μ**	2.85	2.83	2.88	2.82	2.80	2.85	2.95	2.90	3.01	**<.0001**
**Harmfulness of e-cigarettes, %**										
Not at all harmful	3.31	2.88	3.79	3.33	2.84	3.90	3.22	2.45	4.22	
Slightly harmful	9.92	9.17	10.73	10.38	9.43	11.41	8.26	7.07	9.64	
Somewhat harmful	38.27	37.06	39.49	38.84	37.52	40.17	36.19	33.54	38.93	
Very harmful	27.52	26.34	28.72	26.76	25.53	28.02	30.29	27.35	33.40	
Extremely harmful	18.34	17.29	19.43	17.93	16.78	19.13	19.83	17.39	22.53	
Don't know	2.65	2.24	3.14	2.77	2.29	3.35	2.20	1.54	3.13	
**Continuous measure (0 to 4), μ**	2.49	2.46	2.52	2.47	2.44	2.50	2.57	2.51	2.62	**0.0052**
**Harmfulness of nicotine, %**										
Not at all harmful	2.59	2.25	3.00	2.57	2.16	3.05	2.68	1.88	3.82	

Slightly harmful	6.66	6.12	7.24	6.79	6.21	7.42	6.19	4.87	7.82	
Somewhat harmful	25.03	23.86	26.25	25.78	24.56	27.04	22.31	19.57	25.32	
Very harmful	35.87	34.77	36.99	35.80	34.62	37.00	36.15	33.15	39.26	
Extremely harmful	29.63	28.32	30.98	28.91	27.48	30.38	32.28	29.43	35.28	
Don’t know	0.20	0.13	0.33	0.16	0.08	0.29	0.38	0.18	0.82	
**Continuous measure (0 to 4), μ**	2.83	2.81	2.86	2.82	2.79	2.85	2.90	2.83	2.96	**0.0262**
**Harmfulness of nicotine in cigarettes, %**										
Not at all harmful	2.40	2.05	2.81	2.48	2.06	2.97	2.12	1.50	3.01	
Slightly harmful	7.88	7.29	8.52	7.94	7.27	8.66	7.69	6.21	9.50	
Somewhat harmful	28.20	27.02	29.40	28.75	27.57	29.96	26.18	23.69	28.83	
Very harmful	33.81	32.65	34.99	33.82	32.53	35.14	33.78	31.06	36.61	
Extremely harmful	27.41	26.26	28.60	26.71	25.44	28.01	30.00	27.19	32.97	
Don’t know	0.29	0.19	0.45	0.31	0.19	0.51	0.23	0.09	0.58	
**Continuous measure (0 to 4), μ**	2.76	2.74	2.79	2.75	2.72	2.78	2.82	2.76	2.88	**0.0338**
**Harmfulness of nicotine in e-cigarettes, %**										
Not at all harmful	3.02	2.63	3.46	3.14	2.68	3.67	2.57	1.93	3.41	
Slightly harmful	11.81	10.98	12.68	12.16	11.15	13.24	10.53	9.17	12.05	
Somewhat harmful	40.86	39.70	42.03	41.25	39.99	42.51	39.44	36.91	42.02	
Very harmful	25.67	24.73	26.63	24.97	23.81	26.16	28.25	25.65	31.00	
Extremely harmful	16.47	15.43	17.57	16.23	15.02	17.52	17.35	15.09	19.87	
Don’t know	2.18	1.86	2.55	2.26	1.89	2.70	1.87	1.32	2.65	
**Continuous measure (0 to 4), μ**	2.42	2.39	2.44	2.40	2.37	2.43	2.48	2.43	2.54	**0.0131**
**Harmfulness of nicotine in NRT, %**										
Not at all harmful	4.37	3.91	4.89	4.69	4.16	5.29	3.21	2.38	4.31	
Slightly harmful	20.27	19.22	21.37	20.61	19.43	21.85	19.04	16.64	21.69	
Somewhat harmful	41.84	40.41	43.29	41.74	40.22	43.28	42.22	39.00	45.50	
Very harmful	17.59	16.60	18.63	17.03	15.96	18.15	19.66	17.61	21.88	
Extremely harmful	13.88	12.87	14.96	13.94	12.82	15.14	13.66	11.78	15.79	
Don’t know	2.03	1.66	2.49	1.98	1.56	2.52	2.22	1.54	3.19	
**Continuous measure (0 to 4), μ**	2.17	2.14	2.20	2.15	2.12	2.19	2.22	2.16	2.28	**0.0376**
**Harmfulness of LNC versus NNC cigarettes, %**										
Less harmful	13.13	12.13	14.19	13.10	12.02	14.26	13.22	11.15	15.60	
About the same	82.08	80.70	83.38	82.08	80.61	83.47	82.07	79.10	84.70	
More harmful	4.23	3.72	4.82	4.43	3.84	5.10	3.52	2.69	4.60	
Don’t know	0.56	0.31	1.02	0.39	0.26	0.57	1.19	0.38	3.70	
**Misperception: Less harmful/don’t know, %**	13.69	12.61	14.84	13.49	12.39	14.67	14.41	12.02	17.17	0.4873

**Addictiveness of LNC versus NNC cigarettes, %**										
Less addictive	13.24	12.27	14.28	12.40	11.32	13.57	16.32	14.04	18.89	
About the same	82.64	81.61	83.63	83.53	82.33	84.66	79.40	76.74	81.82	
More addictive	3.64	3.19	4.14	3.61	3.12	4.17	3.75	2.82	4.97	
Don’t know	0.48	0.35	0.67	0.47	0.32	0.68	0.53	0.28	1.01	
**Misperception: About the same/more addictive/don’t know, %**	86.76	85.72	87.73	87.60	86.43	88.68	83.68	81.11	85.96	0.0026
**Nicotine in cigarettes causes most of the cancer caused by smoking, %**										
Definitely yes	19.07	18.12	20.06	18.64	17.61	19.71	20.64	18.45	23.01	
Probably yes	44.76	43.22	46.31	45.28	43.68	46.88	42.86	39.65	46.14	
Probably not	28.28	27.07	29.53	28.39	27.00	29.82	27.91	25.57	30.37	
Definitely not	6.21	5.58	6.90	6.22	5.49	7.04	6.16	4.94	7.65	
Don’t know	1.68	1.31	2.15	1.47	1.20	1.80	2.44	1.35	4.37	
**Misperception: Definitely yes/probably yes/don’t know, %**	65.51	64.22	66.77	65.39	63.95	66.81	65.93	63.29	68.48	0.7098
**Nicotine causes people to use tobacco, %**										
Definitely yes	40.36	39.16	41.56	41.24	39.97	42.53	37.11	34.08	40.25	
Probably yes	41.53	40.34	42.73	40.83	39.55	42.12	44.09	40.96	47.27	
Probably not	12.70	11.84	13.61	12.73	11.85	13.66	12.57	10.56	14.91	
Definitely not	4.86	4.38	5.38	4.86	4.36	5.41	4.85	3.79	6.20	
Don’t know	0.56	0.32	0.99	0.34	0.22	0.53	1.37	0.50	3.69	
**Misperception: Definitely not/probably not/don’t know, %**	18.12	17.13	19.15	17.93	16.93	18.98	18.79	16.31	21.56	0.5303
**Harmfulness of snus versus cigarettes, %**										
Less harmful	6.09	5.57	6.65	6.28	5.67	6.94	5.40	4.36	6.67	
About the same	64.12	62.82	65.40	64.50	63.02	65.96	62.70	59.93	65.40	
More harmful	27.21	25.94	28.53	26.60	25.17	28.09	29.44	26.75	32.29	
Don’t know	2.58	2.19	3.04	2.62	2.16	3.17	2.46	1.78	3.38	
**Misperception: Same harm/more harm/don’t know, %**	93.91	93.35	94.43	93.72	93.06	94.33	94.60	93.34	95.64	0.2159
**Harmfulness of e-cigarettes versus cigarettes, %**										
Less harmful	18.69	17.76	19.66	18.72	17.66	19.82	18.60	16.42	20.99	
About the same	70.18	69.01	71.33	69.93	68.58	71.25	71.09	68.43	73.61	
More harmful	8.96	8.16	9.83	9.08	8.14	10.11	8.55	7.16	10.18	
Don’t know	2.17	1.85	2.54	2.28	1.89	2.74	1.77	1.22	2.55	
**Misperception: About the same/more harmful/don’t know, %**	81.31	80.34	82.24	81.28	80.18	82.34	81.40	79.01	83.58	0.9270

Harmfulness of e-cigarettes versus NRT, %										
Less harmful	11.04	10.39	11.73	11.50	10.74	12.29	9.40	7.96	11.06	
About the same	64.61	63.31	65.89	64.61	63.16	66.03	64.62	61.75	67.39	
More harmful	22.09	21.12	23.09	21.69	20.63	22.79	23.55	21.11	26.17	
Don't know	2.26	1.91	2.66	2.21	1.82	2.68	2.44	1.69	3.50	
**Misperception: About the same/less harmful/don't know, %**	77.91	76.91	78.88	78.31	77.21	79.37	76.45	73.83	78.89	0.1827

[1]P-value comparing daily versus some day smokers; LCL: lower 95% confidence interval; UCL: upper 95% confidence interval; HS: High school; LNC: Low nicotine cigarettes; NNC: Normal nicotine cigarettes; NRT: nicotine replacement therapy
